# Relationship between spiritual health and quality of life in patients with T2DM

**DOI:** 10.15649/cuidarte.5149

**Published:** 2026-05-21

**Authors:** Daniel Alberto Valderrama Ruiz, Héctor Riquelme-Heras, Laura Isabel Valderrama Ruiz, Iracema Sierra, Raúl Gutierrez-Herrera

**Affiliations:** 1 Hospital Universitario, Universidad Autónoma de Nuevo León, Monterrey, Mexico. E-mail: drdanielvalderrama@gmail.com Universidad Autónoma de Nuevo León Monterrey Mexico drdanielvalderrama@gmail.com; 2 Hospital Universitario, Universidad Autónoma de Nuevo León, Monterrey, Mexico. E-mail: riquelme@alumni.com Universidad Autónoma de Nuevo León Monterrey Mexico riquelme@alumni.com; 3 Hospital La Carlota, Universidad de Montemorelos, Montemorelos, Mexico. E-mail: laurai.valderrama@gmail.com Universidad de Montemorelos Montemorelos Mexico laurai.valderrama@gmail.com; 4 Hospital Universitario, Universidad Autónoma de Nuevo León, Monterrey, Mexico. E-mail: irasierra@hotmail.com Universidad Autónoma de Nuevo León Monterrey Mexico irasierra@hotmail.com; 5 Hospital Universitario, Universidad Autónoma de Nuevo León, Monterrey, Mexico. E-mail: gutierrezrf@hotmail.com Universidad Autónoma de Nuevo León Monterrey Mexico gutierrezrf@hotmail.com

**Keywords:** Type 2 Diabetes Mellitus, Spirituality, Quality of Life, Health Services, Hospitals, Diabetes Mellitus Tipo 2, Espiritualidad, Calidad de Vida, Servicios de Salud, Hospitales, Diabetes Mellitus Tipo 2, Espiritualidade, Qualidade de Vida, Serviços de Saúde, Hospitais

## Abstract

**Introduction:**

Type 2 diabetes mellitus is a chronic disease that impacts health-related quality of life. Spiritual health may positively influence patients’ well-being, being a relevant aspect for improving comprehensive management.

**Objective:**

To assess the relationship between spiritual health and health-related quality of life in patients with type 2 diabetes mellitus, aiming to provide evidence to support interventions integrating medical and spiritual aspects.

**Materials and Methods:**

A cross-sectional study conducted in 2024 with 230 patients from three hospitals. Spiritual health was measured using the ESE-UM scale, and health-related quality of life with the D-39 questionnaire. Spearman’s correlation and non-parametric tests were used to analyze associations with sociodemographic (age, sex, socioeconomic status) and clinical factors (insulin use, disease progression).

**Results:**

A significant positive correlation was found between spiritual health and health-related quality of life (ρ=0.338, p<0.001). Women and patients with high socioeconomic status reported greater spiritual health, while younger adults and those not using insulin showed better health-related quality of life.

**Discussion:**

The findings confirm that spiritual health serves as a coping resource in type 2 diabetes mellitus, enhancing health-related quality of life, consistent with prior studies. Factors such as sex, socioeconomic status, and hospital setting influenced both variables.

**Conclusion:**

Spiritual health significantly improves health-related quality of life in type 2 diabetes mellitus patients, influenced by sex, socioeconomic status, and hospital environment. Incorporating spiritual care into type 2 diabetes mellitus management could optimize clinical and well-being outcomes, considering these sociodemographic variables.

## Resumen

**Introducción:**

La diabetes mellitus tipo 2 (DM2) es una enfermedad crónica que afecta la calidad de vida relacionada con la salud (CVRS). La salud espiritual puede influir positivamente en el bienestar de los pacientes, siendo un aspecto relevante para mejorar su manejo integral.

**Objetivo:**

Evaluar la relación entre salud espiritual y CVRS en pacientes con DM2, con el propósito de generar evidencia que apoye intervenciones que integren aspectos médicos y espirituales.

**Materiales y métodos:**

Estudio transversal realizado en 2024 con 230 pacientes de tres hospitales. La salud espiritual se midió con la escala ESE-UM y la CVRS con el cuestionario D-39. Se emplearon correlación de Spearman y pruebas no paramétricas para analizar asociaciones con factores sociodemográficos (edad, sexo, nivel socioeconómico) y clínicos (uso de insulina, avance de la enfermedad).

**Resultados:**

Se encontró una correlación positiva significativa entre salud espiritual y CVRS (ρ=0,338, p<0,001). Las mujeres y los pacientes con nivel socioeconómico alto reportaron mayor salud espiritual, mientras que los adultos jóvenes y aquellos sin tratamiento con insulina mostraron mejor CVRS.

**Discusión:**

Los resultados confirman que la salud espiritual actúa como un recurso de afrontamiento en DM2, mejorando la CVRS, en línea con estudios previos. Factores como género, nivel socioeconómico y hospital de atención influyeron en ambas variables.

**Conclusiones:**

La salud espiritual mejora significativamente la CVRS en pacientes con DM2, siendo influenciada por sexo, nivel socioeconómico y entorno hospitalario. Incorporar el cuidado espiritual en el manejo de la DM2 podría optimizar los resultados clínicos y de bienestar, considerando estas variables sociodemográficas.

## Resumo

**Introdução:**

A diabetes mellitus tipo 2 (DM2) é uma doença crônica que afeta a qualidade de vida relacionada à saúde (QVRS). A saúde espiritual pode influenciar positivamente o bem-estar dos pacientes, tornando-se um aspecto relevante para aprimorar seu manejo integral.

**Objetivo:**

Avaliar a relação entre saúde espiritual e QVRS em pacientes com DM2, com o objetivo de gerar evidências para embasar intervenções que integrem aspectos médicos e espirituais.

**Materiais e métodos:**

Foi realizado um estudo transversal em 2024 com 230 pacientes de três hospitais. A saúde espiritual foi mensurada pela escala ESE-UM e a QVRS pelo questionário D-39. O coeficiente de correlação de Spearman e testes não paramétricos foram utilizados para analisar as associações com fatores sociodemográficos (idade, sexo, nível socioeconômico) e clínicos (uso de insulina, progressão da doença).

**Resultados:**

Foi encontrada uma correlação positiva significativa entre saúde espiritual e QVRS (ρ=0,338, p<0,001). Mulheres e pacientes com alto nível socioeconômico relataram melhor saúde espiritual, enquanto adultos jovens e aqueles que não utilizavam insulina apresentaram melhor QVRS.

**Discussão:**

Os resultados confirmam que a saúde espiritual atua como um recurso de enfrentamento no diabetes tipo 2, melhorando a QVRS, em consonância com estudos anteriores. Fatores como sexo, nível socioeconômico e ambiente hospitalar influenciaram ambas as variáveis.

**Conclusões:**

A saúde espiritual melhora significativamente a QVRS em pacientes com diabetes tipo 2 e é influenciada por sexo, nível socioeconômico e ambiente hospitalar. Incorporar o cuidado espiritual no manejo do diabetes tipo 2 pode otimizar os desfechos clínicos e de bem-estar, considerando essas variáveis sociodemográficas.

## Introduction

Diabetes mellitus (DM) comprises a group of heterogeneous metabolic disorders characterized by persistent hyperglycemia, resulting from defects in insulin secretion or action, or both^1^. It constitutes a global health crisis, with a prevalence of 536 million people in 2021 and projections of 783 million by 2045, according to the International Diabetes Federation^2^. In Mexico, DM represents a significant public health challenge, affecting 18.30% of the population (12.60% diagnosed and 5.80% undiagnosed), according to the 2022 National Health and Nutrition Survey^3^. Type 2 diabetes mellitus (T2DM), its most common form, has a multifactorial etiology that integrates genetic and environmental factors, such as obesity, sedentary lifestyle, and consumption of ultra-processed foods^4^. This condition generates short- and long-term complications that negatively affect health-related quality of life (HRQoL), an essential indicator for assessing the impact of the disease and the effectiveness of its management^5^-^7^.

(HRQoL), understood as the subjective perception of the impact of health on physical, psychological, and social well-being, has acquired increasing relevance in modern medicine as a complementary indicator to traditional clinical variables^8^,^9^. The latter, although essential, are insufficient to comprehensively reflect the effects of health interventions and the patient's experience with their illness^10^-^12^. In the context of type 2 diabetes, lower HRQoL has been associated with higher mortality rates^13^, highlighting the need to identify factors that modulate it as priority targets for preventive interventions^14^,^15^.

In this context, spiritual health emerges as a relevant dimension in the management of chronic diseases. Defined as a state of well-being in harmony with the transcendent, the environment, and oneself, according to individual beliefs, spirituality is distinguished from religiosity by its personal and existential character^16^. Koenig conceptualizes spirituality as the personal search for and experience of the sacred or transcendent, an inner process that ranges from inquiry to devotion, while he defines religiosity as an organized system of beliefs, practices, rituals, and norms derived from historical traditions that link the individual to the transcendent and a community of faith^17^.

For his part, Moreira-Almeida describes religiosity as an institutional framework oriented towards facilitating closeness with the sacred, and spirituality as an individual search to understand the ultimate questions about the meaning of life and the relationship with the transcendent, which may or may not manifest itself within a formal religious tradition^18^. Both authors recognize the overlap between both constructs, but agree in reserving their use for phenomena specifically related to the sacred, proposing their joint analysis to evaluate their effects on health.

The assessment of spiritual health in clinical contexts has advanced with the development of validated tools, such as the Spiritual Wellbeing Scale (SWBS), the Functional Assessment of Chronic Illness Therapy-Spiritual Well-Being (FACIT -Sp) and the Montemorelos University Spiritual Health Scale (ESE-UM) were used. The ESE-UM, used in this study, was created in 2017 in Mexico by researchers from the University of Montemorelos and validated in Mexican samples (n=822 local and n=3514 national) with high reliability. Its structure of 39 items in three dimensions - relationship with Supreme Being and spiritual beliefs (14 items, α=0.949), relationship with oneself (12 items, α=0.938), and relationship with others and nature (13 items, α=0.921) - makes it especially suitable for this contex^16^.

Studies have linked spirituality with better health outcomes, greater patient satisfaction, and a higher HRQoL^19^-^21^. In medical crises such as chronic illnesses^22^,^23^, surgical procedures^24^, or end-of-life situations^25^, it acts as a key coping mechanism. There is also evidence that both religion and spirituality can positively influence the management and experience of chronic degenerative diseases such as diabetes^26^-^28^.

In Mexico, where more than 93% of the population professes some religion^29^, spiritual health could have a particularly significant impact. However, its relationship with HRQoL in patients with type 2 diabetes remains understudied.

This study examines the correlation between spiritual HRQoL in patients with type 2 diabetes T2D treated in 2024 at three hospitals in Nuevo León, Mexico: the“Dr. José Eleuterio González” University Hospital (public, academic), La Carlota Hospital (private, affiliated with the Seventh-day Adventist Church), and the Montemorelos General Hospital (public, rural). This analysis aims to contribute to the understanding of how spiritual dimensions can be integrated into the comprehensive care of patients with T2D, improving their HRQoL and overall well-being. It was hypothesized that greater spiritual health would correlate positively with better health-related quality of life.

## Materials and Methods

**Design and participants**

A cross-sectional, empirical, descriptive, and correlational study was conducted between January and October 2024 to analyze the relationship between spiritual health and in 230 patients over 18 years of age diagnosed with T2DM, treated in the outpatient clinics of three hospitals in Nuevo León, Mexico: Montemorelos General Hospital (n=77), La Carlota Hospital (n=77), and "Dr. José Eleuterio González" University Hospital (n=76). Participants were selected using non-probability sampling of consecutive cases, including all patients who met the inclusion criteria (over 18 years of age with T2DM) and excluding those with type 1 diabetes mellitus, severe cognitive impairment, serious mental disorders that affected the understanding of the instruments, or with incomplete data.

The selection of the three hospitals was based on their institutional diversity (level of care, management, and spiritual approach), ensuring representativeness of patients with type 2 diabetes in public, private, and academic settings. The Montemorelos General Hospital, a second-level public hospital affiliated with the Seguro Popular program, primarily serves patients without insurance or resources for private services in rural and semi-urban areas and does not have institutionalized spiritual care services. La Carlota Hospital, a second-level private hospital with a religious affiliation (Seventh-day Adventist Church), integrates spiritual care as an essential component of its care model, offering active chaplaincy and spiritual counseling integrated into the treatment plan. The "Dr. José Eleuterio González" University Hospital, a third-level public academic hospital affiliated with the Autonomous University of Nuevo León (UANL), has an interfaith chaplaincy service available on demand, with multifunctional prayer spaces and spiritual care requested by the patient or family, but it is not systematically integrated into the care protocol.

Although the study was conducted in three hospitals with contrasting institutional characteristics, a formal multicenter coordination structure was not implemented. Standardization of procedures was achieved through training of data collection personnel, use of an identical operating manual, digital instruments in QuestionPro, and supervision by the principal investigator, as well as the application of the instruments in the same order and under the same conditions (private space, iPad, duration ≤30 min).

Since there is no consolidated record of the total number of patients with type 2 diabetes treated in these hospitals during the study period, the total outpatient population was assumed to be unknown. The sample size was calculated using the formula for infinite populations, based on a diabetes prevalence in Mexico of 18.30% (P=0.183, Q=0.817) according to ENSANUT 2022^3^ data, a 95% confidence level (Z=1.96), and a maximum permissible error of 5% (E=0.05).

**Tools**

HRQoL was measured using the Diabetes-39 (D-39) quality of life instrument, previously validated in a Mexican population^30^, adapted from 39 to 36 items after an initial exploratory factor analysis (EFA) that identified three items with communalities less than 0.50, the threshold established as a retention criterion. A final EFA with the remaining 36 items showed a KMO index of 0.937, a significant Bartlett's test of sphericity (p<0.001), communalities ≥0.501, and an explained variance of 63.69%, identifying five factors: energy and mobility (10 items), social burden (9 items), anxiety and worry (7 items), diabetes control (7 items), and sexual functioning (3 items). Internal reliability was high (α=0.958). The responses, obtained on a Likert scale from 1 (“not affected at all”) to 7 (“extremely affected”), were summed by dimension and transformed to a scale of 0 to 100, adjusting the method from the previous validation in Mexico^30^ for the 36-item version, without additional weighting. In the original scale of the D-39 instrument, a higher score indicates poorer HRQoL. For comparative and correlation analyses with spiritual health (measured with the ESE-UM), the scores were recoded so that higher values reflected better HRQoL, to align the direction of both scales (higher score = better status).

Spiritual health was assessed using the Spiritual Health Scale (ESE-UM), previously validated in a Mexican population with an adequate Cronbach's alpha coefficient^16^. This scale comprises 39 items distributed across three domains: relationship with a Supreme Being and spiritual beliefs (14 items), relationship with oneself (12 items), and relationship with others and nature (13 items). This instrument measures the extent to which participants consider their convictions, emotions, attitudes, and behaviors to be aligned with what is good, noble, just, or right in relation to the sacred, themselves, and their environment. The exploratory factor analysis (EFA) confirmed the original 39-item structure without the need for item removal, with a KMO of 0.960, significant Bartlett's test (p<0.001), communalities ≥0.51, and 63.76% of the total variance explained. Responses, on a Likert scale from 0 (“strongly disagree”) to 4 (“strongly agree”), were summed and linearly transformed to a scale of 0 to 100 (higher score=better spiritual health), with high reliability (α=0.975). In addition, sociodemographic (age, sex, marital status, education, socioeconomic level according to INEGI) and clinical (duration of type 2 diabetes, insulin use, BMI) data were collected through a structured survey administered to participants.

**Procedure**

Data was collected between January and October 2024 in the outpatient clinics of the three participating hospitals. Patients were invited to participate during their scheduled appointments after their eligibility was verified according to the inclusion and exclusion criteria. Prior to administering the instruments, the purpose of the study was explained, and written informed consent was obtained. The structured surveys (sociodemographic and clinical data) and the D-39 and ESE-UM instruments were administered on iPads via the QuestionPro platform by trained personnel in a private space within each hospital, with an approximate duration of 30 minutes per participant.

**Statistical analysis**

The data were analyzed using SPSS version 27 software. Descriptive statistics were performed to characterize the sample (means, standard deviations, frequencies, and percentages). The non-normality of the HRQoL and spiritual health variables was determined using the Kolmogorov-Smirnov test. Spearman's rank correlation coefficient was used to examine the correlation between spiritual health and HRQoL (total and by dimension). Mann-Whitney U tests were used to compare two groups (e.g., sex), and Kruskal-Wallis H tests were used for more than two groups (e.g., hospitals). A p-value <0.05 was considered statistically significant. The data were stored in Mendeley Data^31^.

**Ethical considerations**

The principles of the Declaration of Helsinki were followed, ensuring voluntary participation, informed consent, and the right to withdraw at any time without repercussions on medical care. Data were stored in a secure database, accessible only to the research team. The study was reviewed and approved by the Research Committee and the Research Ethics Committee of the “Dr. José Eleuterio González” University Hospital during the session of March 7, 2024, under registration number MF24-00004, and subsequently endorsed by the local committees of the Montemorelos General Hospital and La Carlota Hospital, in compliance with Mexican Official Standard NOM-012-SSA3-2012.

## Results

**Characteristics of the participants**

The sample consisted of 230 patients with T2DM seen in outpatient clinics at three hospitals in Nuevo León, Mexico, during 2024. Sociodemographic variables showed a sex distribution of 125 women (54.35%) and 105 men (45.65 %). Regarding religious affiliation, 155 participants (67.39%) identified as Catholic, 49(21.30%) as Christian, 14(6.09%) as having no religion, 8(3.48%) as Adventist, and 4(1.74%) as Jehovah's Witnesses. In terms of educational level, 59(25.65%) had a bachelor's degree, 58(25.22%) had a secondary school education, 54(23.48%) had a primary school education, 44(19.13%) had a high school education, 8(3.48%) had a postgraduate degree, and 7(3.04%) had no formal schooling. The predominant marital status was married (n=139; 60.43%), followed by single (n=27; 11.74%), widowed (n=25; 10.87%), in a common-law union (n=21; 9.13%), and divorced (n=18; 7.83%). Regarding occupation, 74(32.17%) were homemakers, 50(21.74%) were self-employed, 43(18.70%) were employed, 43(18.70%) were retired, and 20(8.70%) were unemployed. Socioeconomic level, classified according to INEGI (the Mexican National Institute of Statistics and Geography), was middle in 119 cases (51.74%), low in 99(43.04%), and high in 12(5.22%). The most frequent health insurance coverage was Seguro Popular (n=127; 55.22%), followed by IMSS (n=70; 30.43%), other (n=16; 6.96%), ISSSTE (n=15; 6.52%), ISSSTELEON (n=1; 0.43%) and SUSPE (n=1; 0.43%).

Regarding clinical and anthropometric variables, 100 patients (43.48%) were using insulin and 130(56.52%) were not. The distribution of body mass index (BMI) was as follows: overweight in 101(43.91%), normal weight in 66(28.70%), class I obesity in 44(19.13%), class II in 12(5.22%), and class III in 7(3.04%). The distribution across hospitals was balanced: 77 patients (33.47%) at La Carlota Hospital, 77(33.47%) at Montemorelos General Hospital, and 76(33.04%) at the “Dr. José Eleuterio González” University Hospital. The mean age was 57.41 ± 14.55 years, the duration of DM2 was 9.47 ± 7.69 years, the weight was 74.32 ± 14.84 kg, the height was 163.06 ± 9.60 cm, and the BMI was 27.87 ± 4.97 kg/m².

**Levels of spiritual health and HRQoL**

Spiritual health (ESE-UM) had a median score of 91.45 (mean 84.91, SD 16.91) on a scale of 0 to 100 (higher score = better spiritual health). Its domains were: relationship with a Supreme Being (96.43), relationship with others/nature (92.76), and relationship with oneself (87.50).

**Table 1:** Descriptive statistics of spiritual health measured with the ESE-UM scale

Variable	Med [RIC]	SD	Mode	Skewness	Kurtosis
ESE-UM Total	91.45 [77.47;97.37]	16.91	100	-1.578	2.242
Relationship with Supreme Being	96.43 [85.71;100]	18.63	100	-2.128	4.513
Relationship between others and nature	92.76 [81.58;98.68]	16.13	100	-1.704	2.981
Relationship with oneself	87.50 [66.67;97.92]	20.62	100	-1.295	1.408

Med: Median, IQR: Interquartile Range, SD: Standard Deviation, ESE-UM: Montemorelos University Spiritual Health Scale.

The HRQoL (D-39) had, in its original scale, a median score of 24.54, where a higher score indicates worse HRQoL. Its domains were: energy/mobility (25.00), social burden (18.52), diabetes control (26.19), anxiety/worry (39.29), and sexual functioning (0.001).

**Table 2:** Descriptive statistics of health-related quality of life measured with the D-39 scale adapted to 36 items

Variable/Dimension	Med* [RIC]	SD	Mode	Skewness	Kurtosis
D-39 Total Score	24.54 [14.81;39.70]	18.91	10.65	0.824	0.124
Energy and mobility	25.00 [10.00;43.33]	22.38	5.00	0.845	0.160
Social overload	18.52 [8.80;39.35]	22.59	0.00	0.957	0.028
Diabetes management	26.19 [9.52;41.07]	21.44	0.00	0.608	-0.327
Anxiety and worry	39.29 [25.60;54.76]	22.94	35.71	0.304	-0.444
Sexual Function	0.00 [00.00;18.06]	24.58	0.00	1.896	2.859

Med: Median, IQR: Interquartile range, SD: Standard deviation, *Median is on the original D-39 scale (higher score = worse HRQoL (health-related quality of life).

**Correlation analysis**

Spearman's rank correlation analysis, employed due to the non-normality of the variables (Kolmogorov-Smirnov, p<0.001), assessed the relationships between the dimensions of health-related quality of life and those of spiritual health, along with their total scores. For this analysis, the HRQoL (D-39) was recoded average by reversing its original scale (1🡒7, 2🡒6, etc.), so that a higher score indicated better HRQoL, aligning with ESE-UM (higher score = better spiritual health).

Globally, a significant positive correlation was found between the total HRQoL score and spiritual health (ρ=0.338, p<0.01). At the dimensional level, the strongest associations were observed between diabetes control and relationship with others (ρ=0.484, p<0.01), and between social overload and relationship with a higher power (ρ=0.399, p<0.01). Anxiety and worry showed notable correlations with all three spiritual dimensions (ρ=0.244–0.287, p<0.01), while the relationship between sexual functioning and relationship with oneself was not significant (ρ=0.122, p>0.05)^29^.

**Table 3:** Correlations between dimensions and total scores of the D-39 adapted to 36 items and the ESE-UM

Variable/Dimension	Relationship with others	Relationship with self	Relationship with a higher being	Spiritual Health (ESE-UM)
Energy/Mobility	0.247**	0.175*	0.169*	-
Diabetes management	0.484**	0.358**	0.406**	-
Anxiety and worry	0.287**	0.244**	0.285**	-
Social overload	0.451**	0.280**	0.399**	-
Sexual Function	0.205**	0.122	0.184*	-
Health-related quality of life (D-39)	-	-	-	0.338**

ESE-UM: Escala de Salud Espiritual de la Universidad de Montemorelos. D-39: Diabetes-39. Prueba de Rho de Spearman *p<0.05, **p<0.01 (bilateral).

**Differences in spiritual health and health-related quality of life according to sociodemographic, clinical, and institutional factors**

Differences in spiritual health (ESE-UM) and HRQoL (D-39, adjusted to 36 items and recoded so that a higher score indicates better HRQoL) were examined using non-parametric tests, due to the non-normality of the data (Kolmogorov-Smirnov test, p<0.001).

Age, classified according to CONAPO as young adult (25-44 years), mature adult (45-60 years), and older adult (>60 years), excluding the youth group due to its low representation (n=2), showed significant differences in HRQoL (p=0.006), but not in spiritual health (p=0.455). Sex revealed differences in spiritual health (p=0.004), but not in HRQoL (p=0.575). Religion did not show significant differences in either HRQoL (p=0.628) or spiritual health (p=0.661). Education level, excluding the groups with no schooling (n=7) and postgraduate education (n=8) due to their low representation, showed differences in spiritual health (p=0.037) but not in HRQoL (p=0.235). Marital status showed differences in HRQoL (p=0.008), but not in spiritual health (p=0.173). Occupation showed differences in spiritual health (p=0.002), but not in HRQoL (p=0.317). Socioeconomic level showed significant differences in both constructs (HRQoL p=0.002, spiritual health p=0.008). Health insurance coverage showed no differences in either HRQoL (p=0.333) or spiritual health (p=0.235); the ISSSTELEON (n=1) and SUSPE (n=1) groups were excluded due to their small sample size.

**Table 4:** Differences in the medians of HRQoL and spiritual health according to sociodemographic factors

Sociodemographic factor	Median spiritual health [IQR]	p-value	Median HRQoL* [IQR]	p-value
Age		0.455		0.006
Young adult	86.84 [76.32;96.71]		80.56 [70.14; 87.50]	
Mature adult	93.42 [78.29;96.71]		75.93 [65.28;84.72]	
Elderly person	92.76 [75.99;98.03]		69.68 [49.07;84.03]	
Sex		0.004		0.575
Male	86.84 [75.00;95.39]		75.93 [59.03;86.11]	
Female	94.08 [83.22;98.03]		74.54 [61.35;84.72]	
Schooling		0.235		0.235
Primary	94.74 [82.89;98.19]		72.69 [48.38;84.38]	
Secondary	89.80 [79.12;98.03]		75.00[64.47;84.84]	
Preparatory	84.54 [71.22;96.38]		75.24[62.16;83.10]	
Degree	90.13 [78.29;97.37]		78.70 [65.28;87.96]	
Marital status		0.171		0.008
Married	92.76 [82.89;97.37]		76.85 [62.50;84.72]	
Common-law union	93.42 [80.59;98.36]		78.70 [67.60;86.34]	
Single	92.11 [75.33;97.37]		78.24 [56.48;88.89]	
Divorced	82.56 [73.19;94.90]		69.22 [58.10;85.88]	
Widower	89.47 [64.15;96.07]		49.07 [34.26;76.16]	
Occupation		0.002		0.317
Employee	86,18 [73,68;92,76]		78.24 [68.06;87.96]	
Freelance work	82.90 [65.30;94.90]		77.78 [60.99;86.11]	
Unemployed	93.42 [87.01;97.21]		69.68 [51.85;83.34]	
Housewife	94.74 [85.37;98.68]		71.76 [54.51;84.03]	
Retired	94.08 [83.55;98.68]		77.31 [48.15;85.19]	
Socioeconomic level		0.008		0.002
High	95.39 [83.39;99.18]		91.20 [68.06;92.36]	
Half	93.42 [82.89;98.68]		76.85 [65.28;86.11]	
Low	87.50 [71.71;95.39]		71.30 [49.07;81.94]	
Entitlement		0.235		0.333
IMSS	94.74 [78.13;98.68]		75.24 [54.98;86.92]	
ISSSTE	90.13 [63.16;97.37]		77.31 [65.28;92.59]	
Popular Insurance	90.13 [80.92;96.71]		73.61 [59.96;82.97]	
Other	84.21 [69. 24;95.39]		81.48 [74.77;91.55]	

HRQoL: Health-related quality of life. IQR: interquartile range. IMSS: Mexican Social Security Institute. ISSSTE: Institute for Social Security and Services for State Workers. Mann-Whitney U test to compare two groups (e.g., sex) and Kruskal-Wallis H test for more than two (e.g., occupation). *HRQoL Medians are on the recoded D-39 scale (higher score = better HRQoL).

Duration of type 2 diabetes showed differences in HRQoL (p=0.019), but not in spiritual health (p=0.081). Insulin use revealed differences in HRQoL (p<0.001), but not in spiritual health (p=0.261). Body mass index (BMI), grouping obesity classes I, II, and III under "obesity" due to the limited number of cases in class II (n=12) and class III (n=7), showed differences in spiritual health (p=0.006), but not in HRQoL (p=0.605). Finally, the hospital where care was provided showed significant differences in both constructs (HRQoL p=0.034; spiritual health p<0.001).

**Table 5:** Differences in spiritual health and HRQoL according to clinical factors

Clinical factor	Median spiritual health [RIC]	p-value	Median HRQoL* [IQR]	p-value
Duration of diabetes		0.081		0.019
0-5 years	88.82[75.66;97.70]		78.70 [67.83;87.26]	
6-10 years	86.84[70.56;96.71]		71.76 [53.36;81.83]	
>10 years	93.42[86.51;97.70]		73.61 [50.93;82.63]	
Insulin use		0.261		<0.001
Users	89.80[77.13;96.71]		69.91 [52.08;80.56]	
Non-users	92.77[77.14;98.03]		78.47 [64.47;88.55]	
Body mass index (BMI)		0.006		0.605
Normal	89.15 [51.74;88.43]		77.09 [51.74;88.43]	
Overweight	88.16 [63.43;81.94]		73.61 [63.43;81.94]	
Obesity	96.71 [61.57;87.04]		75.93 [61.57;87.04]	
Care Hospital		<0.001		0.034
La Carlota Hospital	97.37 [64.36;91.20]		77.78 [64.36;91.20]	
Montemorelos General Hospital	86.18 [62.50;82.18]		73.61 [62.50;73.61]	
University Hospital	80.60 [49.42;83.80]		73.85 [49.77;83.80]	

HRQoL: Health-related quality of life. IQR: interquartile range. Mann-Whitney U test to compare two groups (e.g., insulin use) and Kruskal-Wallis H test for more than two (e.g., hospitals). * Median HRQoL scores are on the recoded D-39 scale (higher score=better HRQoL).

## Discussion

The present study identified a positive and statistically significant correlation between spiritual health and HRQoL in patients with T2DM (ρ=0.338, p<0.01). This finding reinforces the accumulating evidence that positions spirituality as a key coping resource in chronic diseases, facilitating emotional regulation, therapeutic adherence, and the perception of control over the disease^32^,^33^. From a theoretical perspective, Koenig conceptualizes spirituality as a dynamic system of beliefs and practices that confers meaning and purpose, modulating the response to chronic stress through neuroendocrine (e.g., cortisol reduction) and behavioral (e.g. , greater self-care) pathways^17^. In the context of type 2 diabetes, where oxidative stress and chronic inflammation exacerbate metabolic impairment, spirituality could act as a psychobiological buffer with an impact on overall well-being^34^.

The high overall score in spiritual health (median 91.45) reflects the deep religious cultural influence in Mexico, where 93% of the population reports religious affiliation according to INEGI^27^. The preeminence of the domain "Relationship with a Supreme Being" (median 96.43) highlights the centrality of theistic transcendence in the Mexican worldview, consistent with studies that identify faith in a higher power as a robust predictor of spiritual well-being in Latin populations^34^,^35^.

The difference by sex coincides with studies that associate the female gender with greater spirituality, possibly due to their participation in religious or community networks^36^. High socioeconomic status influenced both variables, suggesting that resources facilitate spiritual practices or resilience^37^. The decline in HRQoL with age reflects the progressive burden of type 2 diabetes^7^, while the stability of spiritual health contrasts with reports of greater spirituality in older adults^38^, perhaps due to the average age of this cohort.

In the clinical setting, insulin use and longer disease duration were associated with worse HRQoL, indicating disease severity^39^. Surprisingly, spiritual health remained unaffected, suggesting its resilience as an existential construct independent of physical decline. The finding of greater spiritual health in obesity aligns with evidence of positive associations between religiosity/spirituality and elevated BMI in culturally religious populations, such as African Americans^40^, and Australian adults^41^. This phenomenon could be interpreted through the lens of compensatory coping: patients with high BMI resort to spirituality to mitigate social stigma and weight-related guilt, promoting emotional resilience and self-compassion instead of immediate behavioral change. This pattern warrants qualitative research in the Mexican context to clarify its sociocultural mechanisms.

Interhospital variation represents one of the most novel contributions: patients at La Carlota Hospital (an Adventist affiliate) exhibited the greatest spiritual health and HRQoL, significantly surpassing the University Hospital and the General Hospital of Montemorelos. This difference is difficult to explain solely by sociodemographic characteristics and likely reflects the institutional model: La Carlota Hospital integrates systematic chaplaincy and a holistic approach, aligned with Adventist health principles^42^. This institutional gradient suggests that environments with structured spiritual integration enhance subjective outcomes such as quality of life and emotional well-being, a phenomenon already documented in oncology, where spiritual support reduces discomfort and improves therapeutic adherence throughout the oncological journey^43^.

At the dimensional level, moderately large, clinically relevant associations were found between specific domains of the D-39 and the spiritual health dimensions measured by the ESE-UM. The diabetes control domain showed the highest correlations with the three spiritual dimensions (ρ=0.484 with “Relationship with Others”, ρ=0.358 with “Relationship with Self”, and ρ=0.406 with “Relationship with a Supreme Being”; p<0.01), suggesting that the subjective perception of control over the disease is closely linked to greater interpersonal integration, a positive sense of self-acceptance, and a deeper spiritual experience.

Similarly, the social overload dimension showed moderate associations with relationships with others (ρ=0.451) and relationships with a Supreme Being (ρ=0.399), which is consistent with the idea that spiritual resources and interpersonal support networks can act as buffers against the stress that diabetes generates in social and family relationships.

The anxiety and worry dimension showed low but constant correlations (ρ=0.244–0.287) with the three dimensions of the ESE-UM, suggesting that spirituality contributes to mitigating the emotional distress associated with the uncertainty and cognitive load of diabetes.

Finally, the sexual functioning dimension showed the weakest correlation of the set, particularly in relation to oneself (ρ=0.122; p>0.05). This result could be due to sociocultural factors, such as low openness in reporting sexual aspects, cultural influences surrounding modesty, and limitations of the instrument itself in this area. Taken together, these patterns suggest that spirituality does not act as a homogeneous and global factor, but rather is differentially related to specific dimensions of quality of life. This opens opportunities to develop more focused interventions, such as promoting practices that strengthen the "Relationship with a Supreme Being" dimension in those who report a low sense of control or high anxiety.

Based on these findings, it becomes pertinent to move towards the systematic integration of spiritual assessment into the routine care of patients with type 2 diabetes. Implementing brief, validated tools in endocrinology or family medicine consultations would allow for the identification of patients with poor spiritual health for timely referral to chaplaincy or spiritual counseling.

Limitations of this study include its cross-sectional design, which prevents inferring causality, and the small size of the subgroups. Even so, the results strengthen the evidence on the role of spirituality in HRQoL in type 2 diabetes and underscore the need for longitudinal studies to clarify the direction of the association and the potential clinical impact of spiritual interventions.

## Conclusions

Spiritual health is significantly correlated with improved HRQoL in patients with T2D in Nuevo León, Mexico, with notable variations by sex, socioeconomic status, and hospital setting in 2024. Women, higher socioeconomic groups, and patients at Hospital La Carlota showed greater spiritual health, while younger age and non-insulin use predicted better HRQoL. Taken together, these findings strengthen the evidence that spiritual health functions as a protective and potentially modifiable resource, whose influence extends beyond conventional metabolic control. Its systematic integration into T2D care, through brief screening, referral to chaplaincy or spiritual counseling, and holistic care models, could optimize not only HRQoL but also therapeutic adherence and patients' emotional well-being.
